# Supplementary material for: Photon- and Singlet-Oxygen-Induced Cis–Trans Isomerization of the Water-Soluble Carotenoid Crocin
Source: Int J Mol Sci. 2023 Jun 28;24(13):10783. doi: 10.3390/ijms241310783 (PMC10341558; doi:10.3390/ijms241310783)
Supplement: Supplementary file 1 [file ijms-24-10783-s001.zip › ijms-2452286-supplementary.pdf]

Supplementary Materials

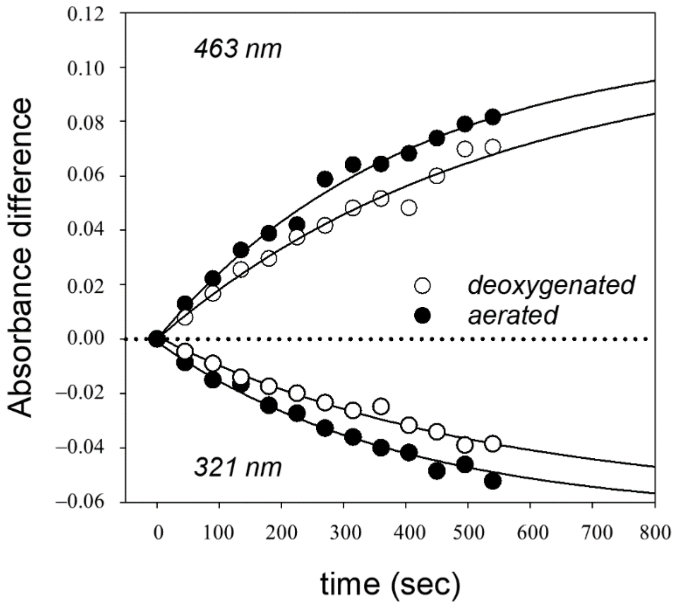

Figure S1. Time evolution of the difference absorbance values at 321 and 463 nm during irradiation at 488 nm, starting from a solution of 13-cis-CR in methanol at the concentration of 3.6 μM. Full line represented the data fitting exponential curves.

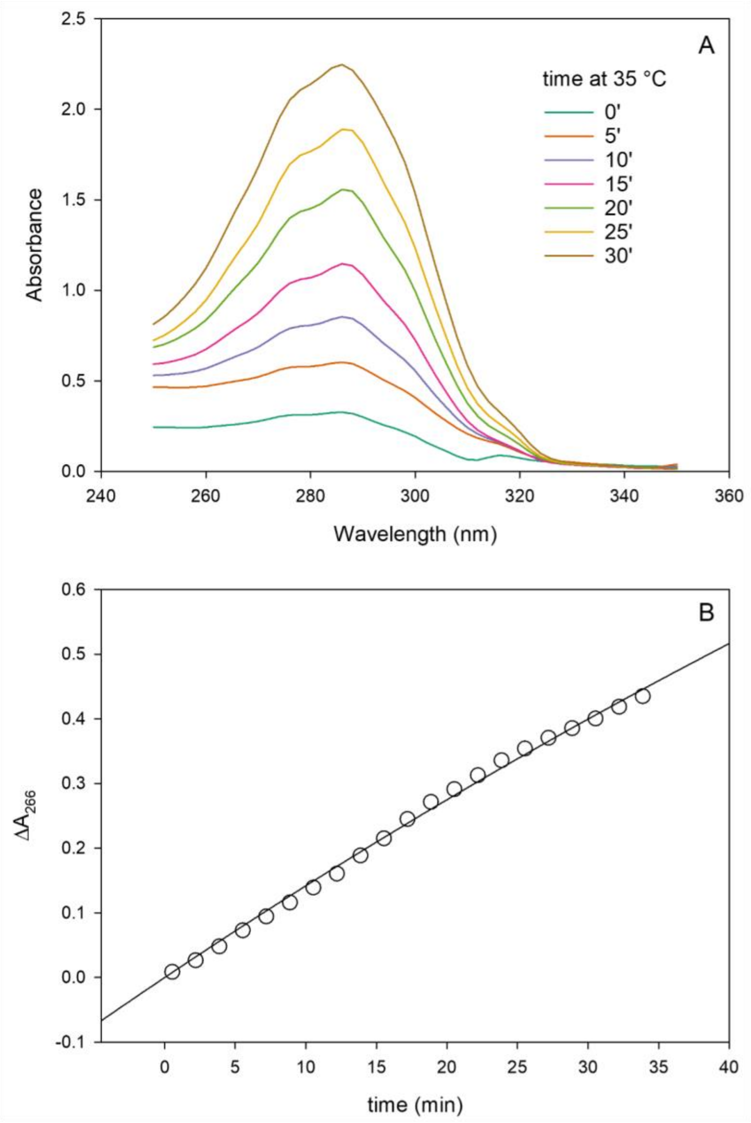

Figure S2. (**A**) Absorption spectra of the naphthalenic acid thermally produced by the decomposition of the 3-(1,4-epidioxy-4-methyl-1,4-dihydro-1-naphthyl)propionic acid at 35 °C. (**B**) Time evolution of the difference absorbance at 266 nm of the naphthalenic acid at 35 °C. The solid line represents the exponential function fitting of data.
